# Influence of Age on Antibody Response and Persistence Following Immunization With MenAfriVac

**DOI:** 10.1093/cid/civ601

**Published:** 2015-11-09

**Authors:** Yuxiao Tang, Brian D. Plikaytis, Marie-Pierre Preziosi, Ray Borrow

**Affiliations:** 1 Meningitis Vaccine Project, PATH, Seattle, Washington; 2 Centers for Disease Control and Prevention, Atlanta, Georgia; 3 Meningitis Vaccine Project, PATH, Ferney-Voltaire, France; 4 Meningitis Vaccine Project, Department of Immunization, Vaccines and Biologicals, World Health Organization, Geneva, Switzerland; 5 Vaccine Evaluation Unit, Public Health England, Royal Manchester Infirmary, United Kingdom

**Keywords:** meningococcal group A conjugate vaccine, MenAfriVac, serum bactericidal antibody, age

## Abstract

***Background.*** A meningococcal group A conjugate vaccine, PsA-TT (MenAfriVac), developed through the Meningitis Vaccine Project and manufactured by the Serum Institute of India, Ltd, was tested in multiple clinical trials conducted mainly in Africa. The impact of age at which subjects were vaccinated on immune response and persistence postimmunization with PsA-TT was the main focus of the current analysis.

***Methods.*** Subjects who were vaccinated with a single dose of 10 µg of PsA-TT at 12–23 months or 22–33 months of age in study A conducted in Mali and The Gambia; at 2–10 years, 11–17 years, or 18–29 years of age in study B conducted in Mali, The Gambia, and Senegal; and at 14–18 weeks, 9–12 months, or 12–18 months of age in study C conducted in Ghana are included in the current analysis. Immunogenicity was measured by group A serum bactericidal antibody (SBA) titer with baby rabbit complement.

***Results.*** Significant differences in SBA titers were found among the age groups in studies B and C both 28 days and 1 year postimmunization. A significant difference in SBA titers between age groups 12–23 months and 22–33 months was only observed 1 year postimmunization in study A. Antibody titers remained at similar levels from 1 to 2 years postimmunization for subjects vaccinated at 12–23 months in study A and at 9–12 months or 12–18 months of age in study C.

***Conclusions.*** Subjects immunized at different ages had different postimmunization immune responses as measured by SBA titers. Toddlers tended to have higher immune responses than infants. This pattern persisted at least 1 year postimmunization.

***Clinical Trials Registration.*** ISRCTN78147026 (study A), ISRCTN87739946 (study B), and ISRCTN82484612 (study C).

A meningococcal group A conjugate vaccine has been developed through the Meningitis Vaccine Project (MVP) to eliminate epidemics of group A *Neisseria meningitidis* in the African sub-Saharan “meningitis belt,” which stretches from Senegal to Ethiopia [1, 2]. MenAfriVac, manufactured by the Serum Institute of India, Ltd (SIIL), consists of 10 µg of meningococcal A polysaccharide-tetanus toxoid (PsA-TT) conjugate vaccine and was prequalified by the World Health Organization (WHO) in June 2010 for use in 1- to 29-year-olds [3]. It is important for policy makers to determine feasible and sustainable immunization strategies to maximize the long-term effectiveness of the vaccine. This paper explores the impact of age effect on meningococcal group A antibody response and persistence following vaccination with a single dose of 10 µg of PsA-TT in individuals between 14 weeks and 29 years of age.

A number of clinical trials were conducted in different locations in West Africa to evaluate the group A conjugate vaccine, PsA-TT. In 3 of these trials, the vaccine was tested in participants with different age ranges. This analysis surveyed these 3 trials and describes the effects of age on the antibody response to the vaccine to support determination of optimal immunization schedules for the vaccine.

## METHODS

The immunogenicity results of a single 10-µg dose of PsA-TT from 3 African trials are investigated. Protocols for the 3 studies have been reported elsewhere [4, Hodgson et al, unpublished data]. The trials were designed and conducted in accordance with the Good Clinical Practice guidelines created by the International Conference on Harmonisation and with the Declaration of Helsinki.

### Overview of the Studies

In study A, healthy toddlers aged 12–23 months were recruited from Mali and The Gambia and randomly assigned to receive either PsA-TT (10 µg), PsACWY (Mencevax ACWY, GlaxoSmithKline), or the *Haemophilus influenzae* type b conjugate Hib-TT (Hiberix, GlaxoSmithKline) in equal proportions at the first vaccination. Ten months later, subjects received a second vaccination with 1 of these 3 vaccines according to a within-group randomization scheme. The detailed design of this study has been presented by Sow et al [4]. The subjects who were vaccinated with a single 10-µg dose of PsA-TT at 12–23 months of age during the first vaccination, and one-third of them who received Hib-TT during the second vaccination, are included in the present study. Subjects who received a single dose of the PsA-TT at 22–33 months of age during the second vaccination following the administration of Hib-TT during the first vaccination are also part of the present study.

In study B, healthy subjects aged 2–29 years were recruited from Mali, The Gambia, and Senegal, evenly stratified into 3 age groups: 2–10 years, 11–17 years, and 18–29 years, and randomly assigned in a ratio of 2:1 to receive the 10 µg of PsA-TT or the PsACWY. The subjects who received PsA-TT in the 3 age groups are part of the current analysis.

In study C, healthy infants of 14–18 weeks of age were recruited from Ghana and randomly assigned to 6 groups, 5 groups where subjects received PsA-TT with different dosages and schedules, concomitantly with vaccines according to the local Expanded Programme on Immunization (EPI) and 1 control group where subjects only received EPI vaccines. There were 3 vaccinations over the course of the study, given at 14–18 weeks, 9–12 months, and 12–18 months of age, respectively. The details of the study design have been described elsewhere by Hodgson et al (unpublished data). The subjects who received a single 10-µg dose of PsA-TT at 14–18 weeks, 9–12 months, or 12–18 months of age are included in the current analysis.

For the subjects who are included in the present study, the age categories prior to vaccination with PsA-TT and vaccine(s) received in each study are provided in Table 1.

**Table 1.** Summary of Vaccine Received, Time of Blood Sample, Follow-up Duration, and Demographics of Study Subjects

Study	Age Category Prior to Immunization	Vaccine Received^a^	Demographics Preimmunization With PsA-TT			28 d Postimmunization With PsA-TT			1 Year Postimmunization With PsA-TT			2 Years Postimmunization With PsA-TT		
			No.^b^	Median Age (Min-Max)	Female, No. (%)	No.^b^	Time of Blood Draw^c^	Mean Duration, mo^d^	No.^b^	Time of Blood Draw^c^	Mean Duration, mo^d^	No.^b^	Time of Blood Draw^c^	Mean Duration, mo^d^
Study A	12–23 mo	1st vac: 10 µg of PsA-TT	201	17 mo (11^e^–23)	92 (45.8)	201	28 d after 1st vac	1.0	65	1 y after 1st vac	14.1	64	Two y after 1st vac	25.7
		2nd vac: Hib-TT												
	22–33 mo	1st vac: Hib-TT	63	25 mo (20–32)	27 (42.9)	62	28 d after 2nd vac	1.0	60	Two years after 1st vac	16.9			
		2nd vac: 10 µg of PsA-TT												
Study B	2–10 y	10 µg of PsA-TT	203	7 y (2–10)	101 (49.8)	203	28 d after vac	1.0	202	1 y after vac	12.6			
	11–17 y	10 µg of PsA-TT	202	14 y (11–17)	75 (37.1)	202	28 d after vac	1.0	196	1 y after vac	12.5			
	18–29 y	10 µg of PsA-TT	199	20 y (18–29)	74 (37.2)	198	28 d after vac	1.0	187	1 y after vac	12.5			
Study C	14–18 wk	1st vac: 10 µg of PsA-TT, EPI	200	14 wk (14–17)	107 (53.5)	200	28 d after 1st vac	0.9						
		2nd vac: EPI												
		3rd vac: EPI												
	9–12 mo	1st vac: EPI	189	9 mo (8–12)	94 (49.7)	187	28 d after 2nd vac	1.0	177	2 y of age	15.3	171	3 y of age	27.1
		2nd vac: 10 µg of PsA-TT, EPI												
		3rd vac: EPI												
	12–18 mo	1st vac: EPI	190	15 mo (12–19)	97 (51.1)	189	28 d after 3rd vac	1.0	183	2 y of age	9.6	181	3 y of age	21.4
		2nd vac: EPI												
		3rd vac: 10 µg of PsA-TT, EPI												

In study A, subjects received 2 vaccinations, one at 12–23 months and the other at 22–33 months of age; in study B, subjects received only 1 vaccination; in study C, subjects received 3 vaccinations, the first at 14–18 weeks, the second at 9–12 months, and the third at 12–18 months of age. The scheduled time of blood draw prior to immunization with PsA-TT in each study was immediately before the immunization.

Abbreviations: EPI, Expanded Programme on Vaccination; Hib-TT, *Haemophilus influenzae* type b–tetanus toxoid; PsA-TT, meningococcal A polysaccharide–tetanus toxoid protein conjugate vaccine; vac, vaccination.

^a^ Vaccine received: the vaccine(s) that subjects who are included in the current study received.

^b^ No.: the number of subjects available for blood draws at a particular time.

^c^ Time of blood draw: the scheduled time of blood draw that was included in current study.

^d^ Mean duration: the actual average follow-up time from vaccination with PsA-TT to blood draw.

^e^ One subject was vaccinated 1 day before his 12-month birthday due to an initial typographical error in his date of birth.

### Immunogenicity Evaluation

This paper analyzes serum bactericidal antibody (SBA) titer to *Neisseria meningitidis* group A capsular polysaccharide, measured by an internationally standardized SBA assay using the standard Centers for Disease Control and Prevention laboratory strain F8238 and baby rabbit complement [5]. The assays were performed at the Vaccine Evaluation Unit, Public Health England (formerly Health Protection Agency), Manchester, United Kingdom. The lower limit of detection for the assay was an SBA titer of 4.

In each of the 3 studies, at maximum 4 blood draws are included in the current study. Blood samples obtained before vaccination with PsA-TT and 28 days after the vaccination are part of this analysis. Contingent upon availability, the blood samples collected at 1 and 2 years, approximately, following the vaccination with PsA-TT are of interest as well. Specifically, for subjects who received PsA-TT at 12–23 months in study A and at 9–12 months or 12–18 months of age in study C, all 4 blood samples were available for this analysis. For subjects who were vaccinated with PsA-TT at 22–33 months in study A and at 2–10 years, 11–17 years, or 18–29 years of age in study B, 3 blood samples (before, 28 days after, and 1 year after vaccination with PsA-TT) were available. Because subjects vaccinated with a single dose of 10 µg of PsA-TT at 14–18 weeks of age in study C received a second dose of PsA-TT at 9–12 months old, only 2 blood samples collected prior to the first dose of PsA-TT and 28 days after the first dose are included in this analysis for these subjects. For the blood samples included in the present analysis, the scheduled times when they were collected in each of the 3 studies are reported in Table 1.

### Statistical Analysis

SBA geometric mean titers (GMTs) and their 95% confidence intervals (CIs) were calculated. SBA GMTs prior to immunization with PsA-TT were compared using *t* test and analysis of variance (ANOVA). SBA GMTs between the age groups within each study at 28 days, 1 year, and 2 years postimmunization were compared using a mixed-effects model adjusted for preimmunization titers, sex, time, and interaction effects of interest with log_2_-transformed titers as outcomes. Reverse cumulative distribution curves of SBA titers are provided at each time point. Analyses were carried out in the intention-to-treat population. Missing data were treated as missing at random. All tests were 2-sided with a significance level of .05. Data analysis was done using SAS software, version 9.1.3.

## RESULTS

### Study Population

In study A, among 201 toddlers aged 12–23 months who received a single dose of 10 µg of PsA-TT at the first vaccination, 65 and 64 are part of this analysis at 1 year and 2 years after the first vaccination, respectively. Among 63 toddlers who were vaccinated with a single 10-µg dose of PsA-TT at 22–33 months of age during the second vaccination, 60 are available for this analysis at 1 year postvaccination with PsA-TT.

In study B, 604 subjects received a single 10 µg dose of PsA-TT at baseline, 203 in the age group 2–10 years, 202 in the age group 11–17 years, and 199 in the age group 18–29 years. Five hundred eighty-five subjects remained 1 year after vaccination, 202 in the age group 2–10 years, 196 aged 11–17 years, and 187 aged 18–29 years.

In study C, 200 subjects received a dose of 10 µg of PsA-TT at the age of 14–18 weeks, 189 subjects were vaccinated with PsA-TT at 9–12 months, and 190 subjects were vaccinated with PsA-TT at 12–18 months of age. Subjects who received a dose of 10 µg of PsA-TT at age 14–18 weeks were vaccinated with another dose of 10 µg of PsA-TT at 9–12 months. The data related to the second dose and subsequent follow-up visits for these subjects are not included in the current analysis. Approximately 1 year after vaccination, 177 subjects vaccinated with PsA-TT at 9–12 months of age and 183 subjects vaccinated with PsA-TT at 12–18 months of age remained in the study. About 2 years after vaccination with PsA-TT, 171 subjects vaccinated at 9–12 months of age and 181 subjects vaccinated at 12–18 months completed the study.

The number of subjects available for the blood draws that were part of the present analysis and the actual average follow-up time from vaccination with PsA-TT to a particular blood draw are summarized in Table 1. The demographics prior to immunization with 10 µg of PsA-TT are presented in Table 1 as well. The youngest subjects were from study C and the oldest participants were from study B.

### SBA Titers Prior to Immunization

**Figure 1.**
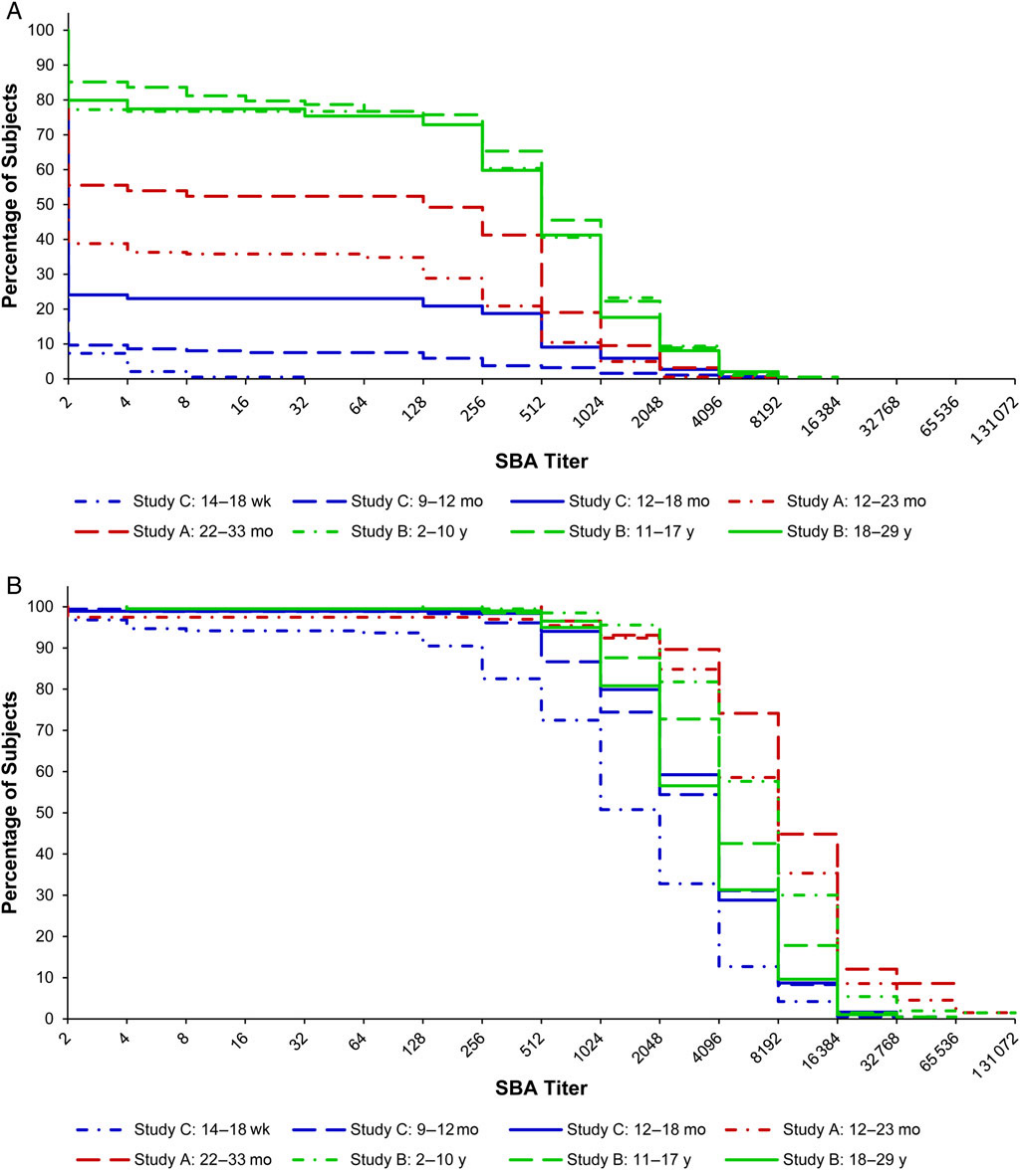
Reverse cumulative distribution curves for serum bactericidal antibody (SBA) titers by follow-up time and age of subjects at immunization. *A*, Prior to immunization. *B*, 28 days after immunization.

SBA GMTs and 95% CIs are presented in Table 2. Reverse cumulative distribution curves of SBA titers are displayed in Figure 1. Prior to immunization, the SBA GMT of the subjects who were to receive PsA-TT at 22–33 months of age was significantly different from that of the subjects who were to be vaccinated with PsA-TT at 12–23 months of age in study A (42.6 [95% CI, 20.3–89.4] vs 14.3 [95% CI, 9.9–20.7]; *P* = .0059). No significant difference in SBA GMT was found among subjects who were scheduled to receive PsA-TT at age of 2–10 years, 11–17 years, and 18–29 years in study B, with GMTs of 209.8 (95% CI, 144.4–304.7), 264.9 (95% CI, 187.7–374.0), and 199.9 (95% CI, 138.5–288.6), respectively. In study C, the GMT for subjects who would receive PsA-TT at age of 12–18 months (7.8 [95% CI, 5.4–11.3]) was significantly different from those who were to be vaccinated at 9–12 months (3.2 [95% CI, 2.5–3.9]) and those scheduled to be vaccinated at 14–18 weeks (2.2 [95% CI, 2.1–2.2]; *P* < .0001 for both comparisons). Subjects who were to receive PsA-TT at the youngest age had the lowest GMT (*P* = .0315 for age group of 14–18 weeks vs age group of 9–12 months). According to Figure 1*A*, subjects in study B seemed to have higher SBA titers than younger subjects in studies A and C prior to immunization with PsA-TT.

**Table 2.** Meningococcal Group A Serum Bactericidal Antibody Geometric Mean Titers

Study	Age Category Prior to Immunization	Preimmunization^a^		28 d Postimmunization^b^		1 Year Postimmunization^c^		2 Years Postimmunization^d^	
		No.	GMT (95% CI)	No.	GMT (95% CI)	No.	GMT (95% CI)	No.	GMT (95% CI)
Study A	12–23 mo	201	14.3 (9.9–20.7)	198	6234.5 (4947.9–7855.7)	65	1035.0 (678.4–1578.9)	64	1313.7 (875.1–1971.9)
	22–33 mo	63	42.6 (20.3–89.4)	58	9342.9 (7043.8–12 392.4)	56	2527.6 (1796.9–3555.5)		
Study B	2–10 y	202	209.8 (144.4–304.7)	203	6743.2 (5900.7–7705.9)	202	3485.9 (3086.4–3937.2)		
	11–17 y	202	264.9 (187.7–374.0)	202	4618.7 (4029.4–5294.2)	196	3075.8 (2641.6–3581.3)		
	18–29 y	199	199.9 (138.5–288.6)	198	3331.6 (2872.3–3864.4)	186	2206.5 (1837.6–2649.4)		
Study C	14–18 wk	191	2.2 (2.1–2.2)	189	1138.9 (866.3–1497.3)				
	9–12 mo	186	3.2 (2.5–3.9)	180	2776.2 (2290.7–3364.6)	175	365.6 (259.2–515.8)	170	481.6 (344.9–672.5)
	12–18 mo	187	7.8 (5.4–11.3)	184	3170.4 (2656.6–3783.6)	179	786.9 (595.2–1040.5)	179	827.6 (610.1–1122.5)

Except for the preimmunization comparisons, the rest of the comparisons were using mixed-effect models. The outcome variables were log_2_-transformed titers. Age category prior to immunization and time (visit points) were the main fixed effects in the models. Study subject was the random effect. The baseline titer, sex, and interaction effects of baseline titer-by-age category, sex-by-age category, baseline titer-by-time, and time-by-age category were also included in the models.

Abbreviations: CI, confidence interval; GMT, geometric mean titer.

^a^ Prior to immunization with PsA-TT: *P* = .0059 for the comparison of SBA GMTs between subjects vaccinated at 12–23 months and 22–33 months of age in study A using *t* test; *P* < .0001 for the comparisons between subjects vaccinated at 14–18 weeks and 12–18 months of age and between subjects vaccinated at 9–12 months and 12–18 months of age; and *P* = .0315 for the comparison between subjects vaccinated at 14–18 weeks and 9–12 months of age in study C using analysis of variance.

^b^ At 28 days postimmunization with PsA-TT: *P* = .0003 for the comparison of SBA GMTs between subjects vaccinated at 2–10 years and 11–17 years of age, *P* < .0001 for the comparison between subjects vaccinated at 2–10 years and 18–29 years of age, *P* = .0023 for comparison between subjects vaccinated at 11–17 years and 18–29 years of age in study B; *P* < .0001 for the comparison between subjects vaccinated at 14–18 weeks and 9–12 months of age and between 14–18 weeks and 12–18 months of age in study C.

^c^ One year postimmunization with PsA-TT: *P* = .0022 for the comparison of SBA GMTs between subjects vaccinated at 12–23 months and 22–33 months of age in study A; *P* < .0001 for the comparison between subjects vaccinated at 2–10 years and 18–29 years of age and *P* = .0038 for the comparison between subjects vaccinated at 11–17 years and 18–29 years of age in study B; *P* = .0026 for the comparison between subjects vaccinated at 9–12 months and 12–18 months of age in study C.

^d^ Two years postimmunization with PsA-TT: *P* = .0282 for the comparison of SBA GMTs between subjects vaccinated at 9–12 months and 12–18 months of age in study C.

**Figure:**
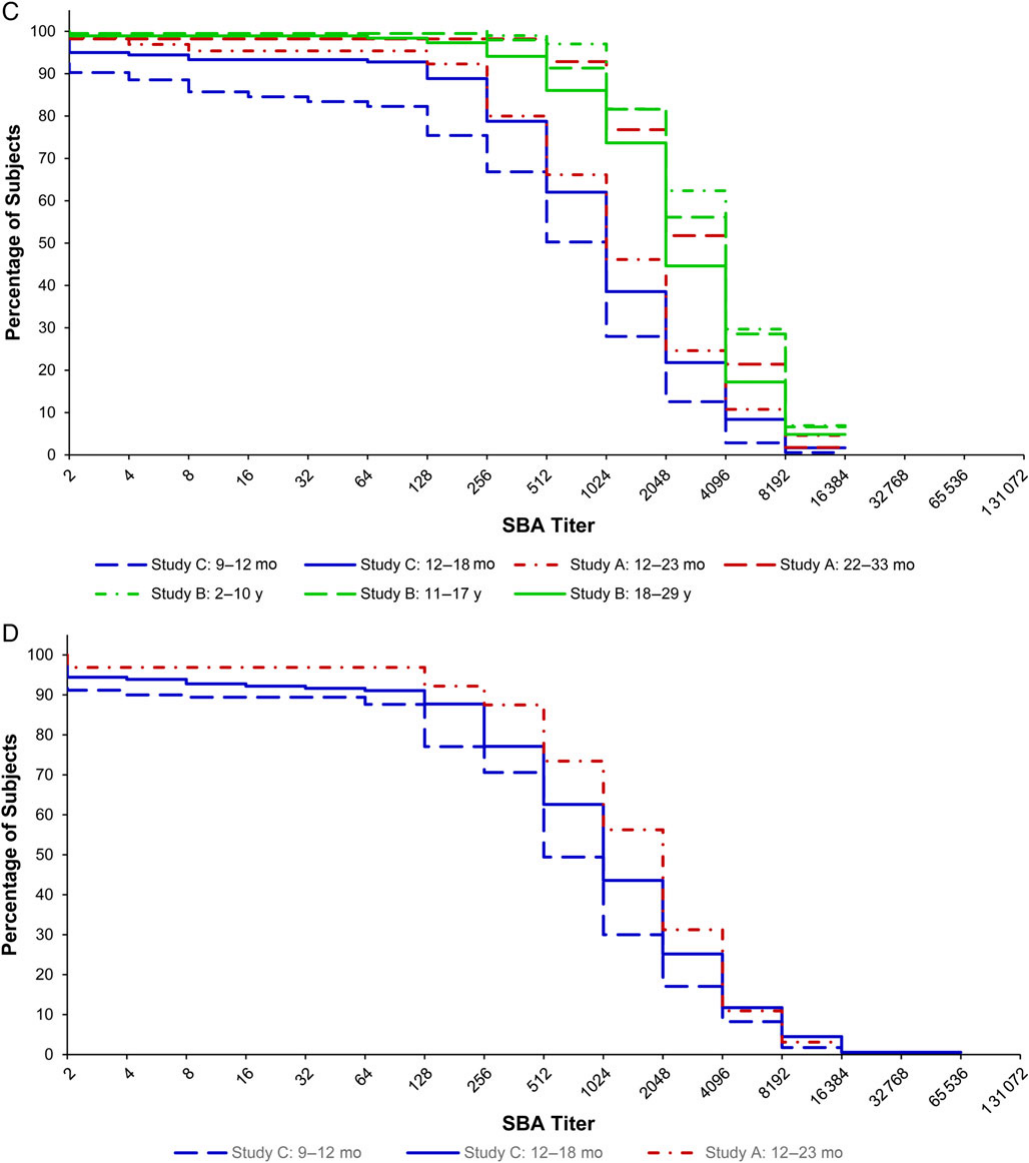
*C*, One year after immunization. *D*, Two years after immunization.

### SBA Titers 28 Days Postimmunization

At 28 days postimmunization with a single dose of 10 µg of PsA-TT, the SBA GMT was 6234.5 (95% CI, 4947.9–7855.7) for subjects 12–23 months of age and 9342.9 (95% CI, 7043.8–12 392.4) for those at 22–33 months of age in study A. The difference in GMTs between these 2 age groups was not statistically significant after adjusting for preimmunization SBA titers, sex, and time. In study B, subjects who were vaccinated at younger ages had greater SBA GMTs 1 month after vaccination with GMTs of 6743.2 (95% CI, 5900.7–7705.9) for 2- to 10-year-olds, 4618.7 (95% CI, 4029.4–5294.2) for 11- to 17-year-olds, and 3331.6 (95% CI, 2872.3–3864.4) for 18- to 29-year-olds. These means were significantly different from each other, with *P* = .0003 comparing 2–10 years vs 11–17 years, *P* < .0001 for 2–10 years vs 18–29 years, and *P* = .0023 for 11–17 years vs 18–29 years. In study C, subjects vaccinated at age of 14–18 weeks had the lowest GMT among the 3 age groups (1138.9 [95% CI, 866.3–1497.3]), compared with 2776.2 (95% CI, 2290.7–3364.6) in those aged 9–12 months, and 3170.4 (95% CI, 2656.6–3783.6) in those aged 12–18 months. The GMT for the 14- to 18-week-olds was significantly different than the GMTs of those aged 9–12 months and 12–18 months (*P* < .0001). The SBA GMT of subjects vaccinated at 9–12 months was not significantly different from that of subjects vaccinated at 12–18 months of age 1 month after vaccination. As shown in Figure 1*B*, subjects in study A tended to have higher SBA titers than subjects in studies B and C 28 days postimmunization with PsA-TT.

### SBA Titers 1 Year and 2 Years Postimmunization

One year following vaccination with a single dose of 10 µg of PsA-TT, the SBA GMT of subjects vaccinated at 22–33 months was significantly different than those immunized at 12–23 months of age in study A (2527.6 [95% CI, 1796.9–3555.5] vs 1035.0 [95% CI, 678.4–1578.9]; *P* = .0022). In study B, the SBA GMTs of the younger subjects were greater than the older participants. Those aged 2–10 years had a GMT of 3485.9 (95% CI, 3086.4–3937.2), those 11–17 years had a GMT of 3075.8 (95% CI, 2641.6–3581.3), and those 18–29 years had a GMT of 2206.5 (95% CI, 1837.6–2649.4). The GMT for the 18- to 29-year-olds was significantly different than that of the 11- to 17-year-olds (*P* = .0038) and 2- to 10-year-olds (*P* < .0001). In study C (on average 15 months following vaccination at 9–12 months and 9 months following vaccination 12–18 months of age), the SBA GMT of subjects who received PsA-TT at 12–18 months was significantly different than that of subjects vaccinated at 9–12 months of age (786.9 [95% CI, 595.2–1040.5] vs 365.6 [95% CI, 259.2–515.8]; *P* = .0026). The pattern shown in Figure 1*C* corroborates the above results. Figure 1*C* also indicates that the cumulative distribution of SBA titers of subjects vaccinated at 12–23 months of age in study A resembled those of subjects vaccinated at 12–18 months of age in study C and were lower than those of subjects in study B at 1 year postimmunization.

Two years after vaccination with a single dose of 10 µg of PsA-TT, the SBA GMT of subjects vaccinated at 12–23 months was 1313.7 (95% CI, 875.1–1971.9) in study A. This was not significantly different than the GMT of this same age group 1 year postimmunization after adjusting for preimmunization SBA titers, sex, and time. In study C (on average 27 months following vaccination at 9–12 months and 21 months following vaccination at 12–18 months of age), the GMT of subjects vaccinated at 12–18 months was significantly different from that of subjects vaccinated at 9–12 months of age (827.6 [95% CI, 610.1–1122.5] vs 481.6 [95% CI, 344.9–672.5]; *P* = .0282). The SBA GMTs remained at similar levels from 1 year to 2 years postimmunization for subjects vaccinated at different ages. Figure 1*D* shows that subjects who were vaccinated at younger ages tended to have lower SBA titers 2 years after vaccination.

## DISCUSSION

This article demonstrates the impact of age on immune response and persistence following immunization with PsA-TT (MenAfriVac). Prior to immunization, subjects who were to receive PsA-TT at the youngest ages (14–18 weeks, 9–12 months, and 12–18 months) from study C had the lowest SBA GMTs, whereas subjects in the oldest age groups (2–10, 11–17, and 18–29 years) from study B had the highest SBA GMTs. A similar pattern was observed within each of studies A and C where participants were younger as well, with the younger subjects showing lower SBA GMTs than the older participants, which is a reflection of an increase in natural immunity with age due to the endemic nature of *N. meningitidis* group A in sub-Saharan Africa and also through exposure to bacteria whose capsules share common moieties with the group A meningococcal capsule [6–8]. As age increased, levels of natural immunity, as measured by SBA GMTs, remained at similar levels in the age groups 2–10, 11–17, and 18–29 years.

One month after vaccination with MenAfriVac, the youngest age group of subjects, 2–10 years, had the greatest SBA GMTs in study B. However, the opposite trend was observed in study C; the subjects vaccinated at 14–18 weeks had the lowest postimmunization SBA GMT among the 3 age groups. Most of the subjects in study A tended to have higher 1-month postimmunization SBA titers than subjects in studies B and C. There are a number of possibilities as to why the 2- to 10-year-olds had the highest SBA GMTs in study B. First, this could be because, with increasing age, the likelihood of exposure to polysaccharide also increases. This exposure can either be positive in the induction of functional SBAs or detrimental in the induction of hyporesponsiveness. Hyporesponsiveness or lower antibody responses have been documented following multiple doses of polysaccharide vaccine, and also lower responses to conjugate vaccine when previously exposed to polysaccharide vaccine [9–11]. Second, this observation may be due to the induction of different quantities of immunoglobulin G (IgG) subclasses that have different efficacy in fixing complement and thus induce different levels of SBA activity. IgG1 and IgG3, as opposed to IgG2, are the most effective of the subclasses in fixing complement. Of most interest is that IgG3 has a greater ability to activate complement than IgG1, has a shorter half-life, and may be present at higher levels in infants and young children than in older children [12].

At 1 year postimmunization, it was shown that, among the 3 studies, subjects who received PsA-TT at the youngest ages in study C still had lowest SBA titers. Within each of the 2 studies with younger participants, subjects vaccinated at younger ages had lower SBA titers than subjects vaccinated at older ages. A considerable drop in SBA GMTs from 28 days to 1 year after vaccination was observed across all age groups, with a larger drop in the younger subjects within each study (83.4% drop in those aged 12–23 months vs 72.9% in age 22–33 months in study A; 48.3% drop in age 2–10 years vs 33.4% in 11–17 years and 33.8% in 18–29 years in study B; 86.8% drop in age 9–12 months vs 75.2% in age 12–18 months in study C). There were limited data available 2 years postimmunization. The SBA GMTs remained at the similar level from 1 year to 2 years postimmunization for subjects vaccinated at 9–12 months and 12–18 months of age in study C and subjects vaccinated at 12–23 months of age in study A. This has demonstrated that immune response to vaccination dropped quickly from peak level at 28 days postimmunization to plateau at 1 year through to 2 years postvaccination.

It is recognized that there was 6 months’ difference in average follow-up time from vaccination with PsA-TT to the last 2 blood samples between subjects vaccinated at 9–12 months and 12–18 months of age in study C. A further investigation is warranted to determine whether or not this difference contributed largely to the observed difference in GMT at 1 year and 2 years postimmunization between the 2 groups of subjects in study C. It is unknown whether other antigens received in study A and in study C affected antibody response induced by PsA-TT or not. Further research is also needed.

After vaccination with MenAfriVac, it was shown that toddlers tended to have greater immune response than infants. In addition, an early sharp antibody decline was observed, with more rapid waning in infants than in toddlers, reaching lower levels 1 year postvaccination. No further decline was observed at 2 years postvaccination, and SBA titers remained sustained in both infants and toddlers.

The results presented in this study, along with epidemiological evidence and programmatic considerations, support the WHO recommendations for the optimal age for routine immunization with meningococcal group A conjugate vaccine: a 1-dose schedule at 9–18 months of age [13]. Documentation of long-term persistence of the immune response and determination of correlates of protection are needed.
